# Membrane vesicle engineering with “*à la carte*” bacterial‐immunogenic molecules for organism‐free plant vaccination

**DOI:** 10.1111/1751-7915.14323

**Published:** 2023-08-02

**Authors:** Irene Jiménez‐Guerrero, Francisco Javier López‐Baena, José Manuel Borrero‐de Acuña, Francisco Pérez‐Montaño

**Affiliations:** ^1^ Department of Microbiology University of Seville Seville Spain

## Abstract

The United Nations heralds a world population exponential increase exceeding 9.7 billion by 2050. This poses the challenge of covering the nutritional needs of an overpopulated world by the hand of preserving the environment. Extensive agriculture practices harnessed the employment of fertilizers and pesticides to boost crop productivity and prevent economic and harvest yield losses attributed to plagues and diseases. Unfortunately, the concomitant hazardous effects stemmed from such agriculture techniques are cumbersome, that is, biodiversity loss, soils and waters contaminations, and human and animal poisoning. Hence, the so‐called ‘green agriculture’ research revolves around designing novel biopesticides and plant growth‐promoting bio‐agents to the end of curbing the detrimental effects. In this field, microbe–plant interactions studies offer multiple possibilities for reshaping the plant holobiont physiology to its benefit. Along these lines, bacterial extracellular membrane vesicles emerge as an appealing molecular tool to capitalize on. These nanoparticles convey a manifold of molecules that mediate intricate bacteria–plant interactions including plant immunomodulation. Herein, we bring into the spotlight bacterial extracellular membrane vesicle engineering to encase immunomodulatory effectors into their cargo for their application as biocontrol agents. The overarching goal is achieving plant priming by deploying its innate immune responses thereby preventing upcoming infections.

## Graphical abstract

In this mini review, we bring into the spotlight bacterial extracellular membrane vesicle engineering to encase bacterial immunomodulatory effectors into their cargo for their application as biocontrol agents. The overarching goal is achieving plant priming by deploying its innate immune responses thereby preventing upcoming infections.

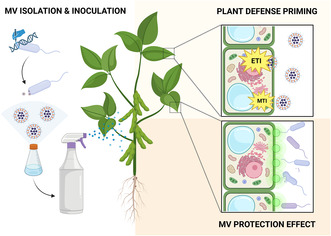

## INTRODUCTION

**FIGURE 1:**
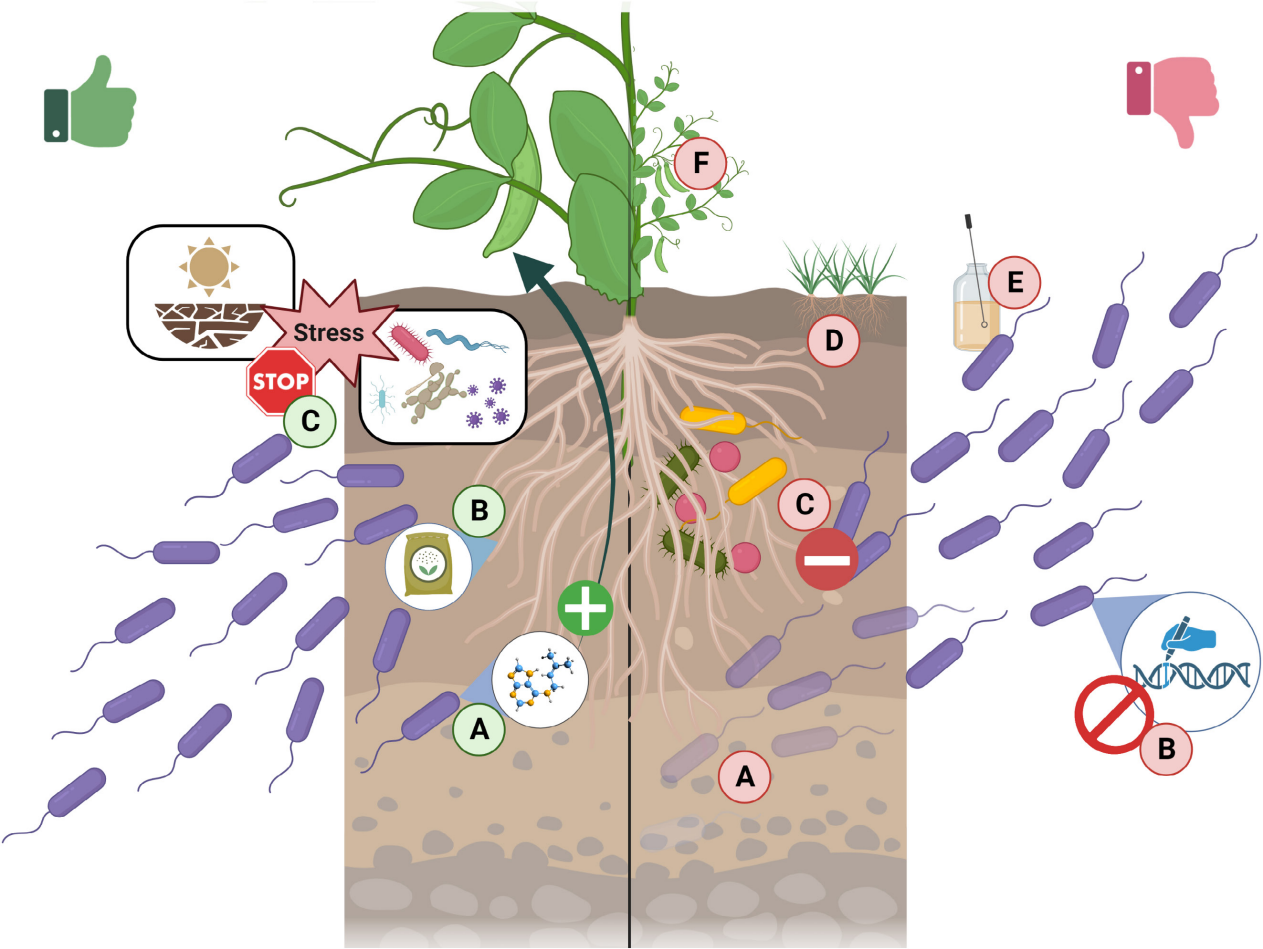
Schematic view representing the main profits and challenges of using plant growth‐promoting rhizobacteria (PGPR). Plant‐beneficial functions include nutrient acquisition (A, left), stimulation of plant growth (B, left), and plant tolerance to multiple abiotic and biotic stresses (C, left). PGPR drawbacks include low persistence in soils (A, right), governmental restrictions to the use of genetically modified microorganisms (B, right), poorer adaptation to rhizosphere (C, right), growth enhancement of non‐target plants (D, right), attenuation of PGPR traits due to non‐favourable environments (F, right) and suboptimized inoculation methodology (E, right). Created with BioRender.com.

World population will exceed 9.7 billion by 2050 before reaching 10.8 billion around 2080. To meet food security in a climate change scenario, which translates into higher frequencies of devastating phenomena, such as droughts, floods and pathogen outbreaks, agricultural productivity must increase by up to 70% (Hunter et al., 2017; van Dijk et al., 2021). Lately, the use of pesticides and herbicides, synthetic fertilizers, and improved plant cultivars, significantly boosted the global crop yield, reducing the risk of global hunger and poverty (Tilman et al., 2002, 2011). However, the massive use of agrochemicals and the application of aggressive practices brings about undesirable detrimental environmental consequences, including chemical runoff, increased pollution, biodiversity losses, and soil degradation (Carvalho, 2006). In the recent years, plant growth‐promoting rhizobacteria (PGPR) have emerged as a crucial component for sustainable agriculture to promote the prophylaxis and therapy of crop‐associated soils, ensuring agricultural sustainability by substituting the roles of agrochemicals to enhance primary food production (Batista & Singh, 2021; Hu et al., 2022; Mitter et al., 2021; Timmis & Ramos, 2021). PGPR can perform a wide range of life‐beneficial functions, including nutrient acquisition, stimulation of plant growth, and plant tolerance to multiple abiotic and biotic stresses (Pérez‐Montaño et al., 2014; Singh et al., 2020). However, while some PGPR have already been successfully commercialized improving crop yields (Díaz‐Zorita & Fernández‐Canigia, 2009; Dobbelaere et al., 2001; O'Hanlon, 2019), functionality and persistence of beneficial microbes as inoculants for important food‐crops is shadowed due to lower persistence in soils and suboptimal rhizosphere colonization abilities with better‐adapted indigenous microbes. Besides, undesirable down‐regulation of plant growth promotion traits to conserve energy and resources, promiscuous host‐specificity that can enhance the growth of wild or invasive plant species and underdeveloped inoculation strategies that penalize also restrict the effective use of PGPR (Haskett et al., 2021; Ofek et al., 2014) (Figure 1). Interestingly, many plant growth‐promoting (PGP) mechanisms that include nitrogen‐fixation, phosphate solubilization, phytohormone production, degradation of xenobiotic pollutants, and biocontrol of pathogenic agents (antibiosis, competition or plant immune system activation) have been enough studied to be genetically engineered and transferred into selected rhizobacterial ‘chassis’, which might suppose the first approach for coping PGPR inconsistency improving crop yields (Haskett et al., 2021; Hu et al., 2022). However, the use of genetically modified rhizobacteria remains a significant public concern, and at this moment, release of engineered microorganisms in most countries is strictly regulated or directly forbidden (Lee, 2010). Thus, there is growing interest in developing novel PGP‐based technologies to harness the beneficial plant–microbe traits and sustainably promote crop performance without the use of living microorganisms, especially under the current constrained conditions consequence of global climate change.

In this review, we discuss the use of tailored extracellular membrane vesicles as microorganism‐free bioagents to tackle ecological and biological limitations of natural PGPR in agriculture. As an example, we propose a strategy to engineer these nano‐conveyors “*à la carte*” in the broad host range pathogenic bacterium *Pseudomonas syringae* using immunogenic agents to vaccinate plants in a biocontrol strategy to prevent subsequent crop infestation.

## PLANT IMMUNE SYSTEM: MICROBE‐ASSOCIATED MOLECULAR PATTERN – AND EFFECTOR‐TRIGGERED IMMUNITIES

**FIGURE 2:**
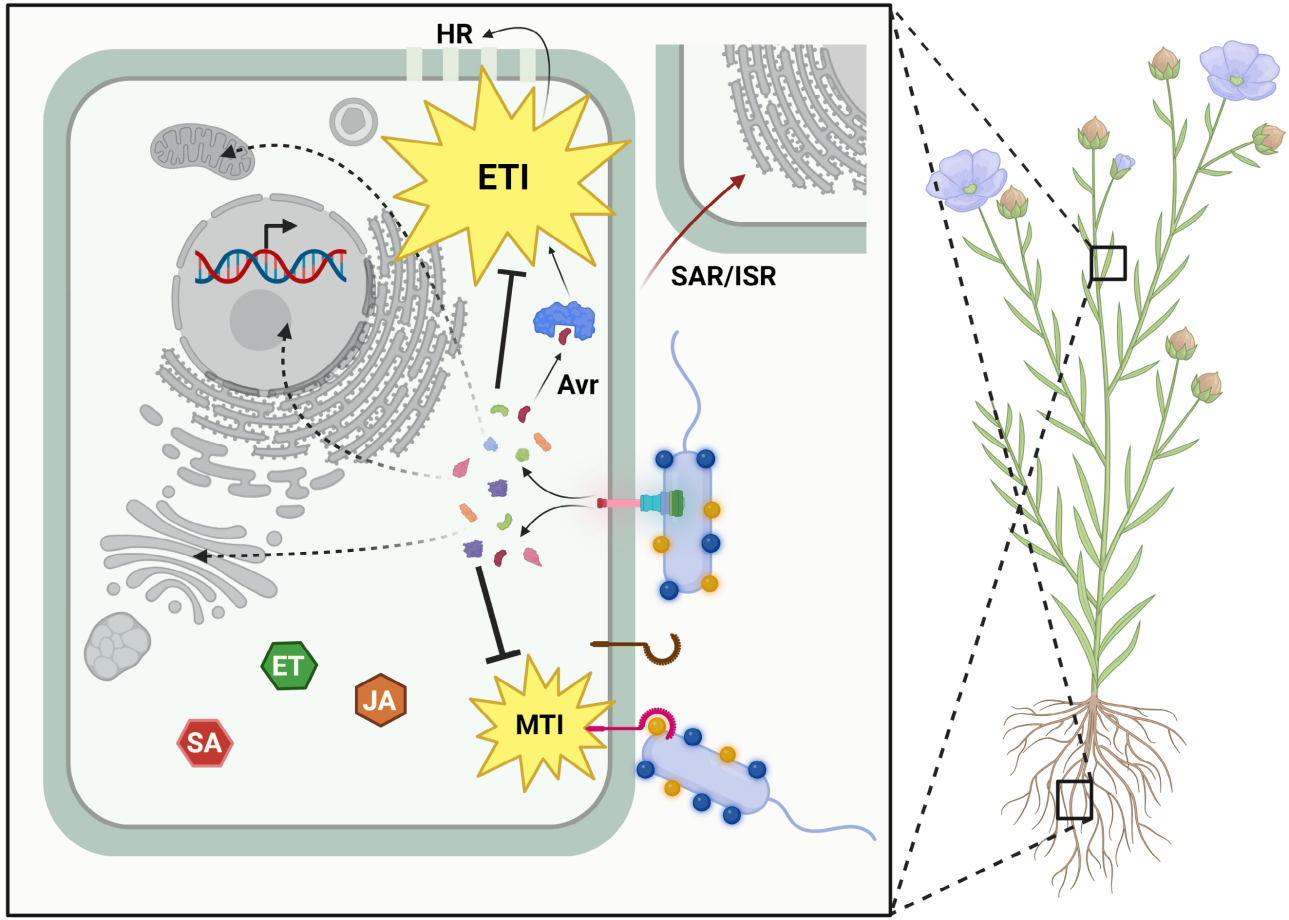
Conceptual illustration of the plant immune system. Microbe‐associated molecular patterns (MAMP) perception results in MAMP‐triggered immunity (MTI), an array of defence responses that counteracts pathogenic infections. Bacterial type III secretion system effectors are directly translocated into the host cell suppressing MTI responses to promote virulence. Conversely, plants recognize certain effectors by avirulence proteins (Avr), eliciting an expanded defence reaction termed effector‐triggered immunity (ETI), which often entails a hypersensitive response (HR). This local response can also be transmitted systemically through systemic acquired resistance (SAR), mediated by salicylic acid (SA), or induced systemic resistance (ISR), often jasmonic acid‐ and ethylene‐dependent (JA and ETI), which primes plants for a more efficient control of upcoming infections. Created with BioRender.com.

Plants utilize a forefront defence barrier against pathogen infection via recognition of the so‐called microbe‐ or pathogen‐associated molecular patterns (MAMPs or PAMPs) by cell‐surface receptors termed pattern recognition receptors (PRRs). For the sake of language economy, we will hereafter name them as microbe–associated molecular patterns: MAMPs. These immunogenic molecules are essential structures for microbes, and for that reason, they are well conserved among pathogenic, saprophytic, and beneficial microorganisms (Newman et al., 2013). With exceptions, MAMPs are essentially parts of the bacterial surface components, encompassing mainly protein (flagellins, xylanases, lectins, or elongation factor Tu) or polysaccharide chemical natures (lipopolysaccharides [LPS], beta‐glycans, chitins or peptidoglycans) (Felix et al., 1993; Gómez‐Gómez et al., 2001; Gust et al., 2007; Mateos et al., 1997; Newman et al., 1995; Ron & Avni, 2004; Umemoto et al., 1997). Their perception results in MAMP‐triggered immunity (MTI), an array of defence responses that can arrest infection of most potential pathogens (Dangl & Jones, 2006). MAMP‐induced responses encompass a plethora of localized mechanisms such as oxidative burst by production of reactive oxygen and nitrogen species, alterations in the plant cell wall, including callose deposition and induction of antimicrobial compounds (Newman et al., 2013). Conversely, in the plant–pathogen warfare, pathogenic microbes utilize a specialized apparatus for protein secretion, the type III secretion system (T3SS) to deliver protein effectors into the host cell promoting virulence through alteration of its metabolism and/or suppression of MTI, which results in effector‐triggered susceptibility (Feng & Zhou, 2012; Macho & Zipfel, 2015). Thus, while the contribution of individual type III effectors (T3Es) to virulence may be subtle, collectively they are generally required for pathogenicity. As countermeasure, plants have evolved to recognize some effectors by concomitant disease resistance (R) proteins that mostly belong to the nucleotide‐binding leucine‐rich repeat family of immune receptors (NLRs) (Duxbury et al., 2016). Thus, upon this specific effector recognition, the R proteins elicit an extensive defence response called effector‐triggered immunity (ETI) that in most cases, blocks the infective process. In this case, the effector is referred to as an avirulence (Avr) protein since the plant–pathogen interaction is incompatible (Dangl & Jones, 2006; Flor, 1971). ETI responses generally entails a hypersensitive response (HR) in host resistant species or non‐host plants, which halts the pathogen with rapid, localized cell death around the infection site owing to transcriptional reprogramming, ion fluxes, massive oxidative burst, lipid peroxidation, and cell wall fortification (Balint‐Kurti, 2019; Mansfield, 2009) (Figure 2). Interestingly, increasing evidence suggests that both signalling branches are functionally connected, being in some cases, simultaneously activated by cell‐surface PRRs and intracellular NLRs, respectively, which results in synergistic and expanded defence responses against pathogens (Ngou et al., 2021).

It is well known that plants can be primed for more efficient activation of further defence responses. For instance, the HR can be induced more efficiently in plants previously subjected to a pathogenic attack resulting in the attenuation of the necrotic lesions (Mauch‐Mani et al., 2017). This plant priming can be activated through different plant responses, such as the systemic acquired resistance (SAR) or induced systemic resistance (ISR), among others. SAR is developed in response to a pathogenic local infection and usually requires the involvement of the plant hormone salicylic acid and its receptor NPR1 (Saleem et al., 2021). ISR is activated in response to root colonization by beneficial microbes and triggered responses are usually jasmonic acid‐ and ethylene‐dependent (Yu et al., 2022). Although priming is reversible, it can be maintained during different stages of the plant's life cycle or even be transmitted to the offspring (Mauch‐Mani et al., 2017). In addition, priming is not only developed locally but can also be transmitted systemically, offering plant protection in plant tissues far off from the initial infection site (Figure 2). In some cases, the activation of these defence responses can even be transmitted to other plants via volatile organic compounds (Brosset & Blande, 2022).

In summary, MAMPs and T3Es play a dual role in the interactions between many plant pathogenic bacteria and plants: while they are required for bacterial surveillance or collectively promote virulence on susceptible plants, some may induce strong defence responses in plants, providing a long‐term immunity or priming for upcoming infective processes. Thus, these host‐determinant immunogenic molecules might be hypothetically used as specific antigens for the development of plant vaccines against one or more pathogenic bacteria.

## TYPE III EFFECTORS OF THE PLANT PATHOGENIC BACTERIUM *PSEUDOMONAS SYRINGAE*: A CASE‐STUDY OF HOST‐DETERMINANT MOLECULES

*Pseudomonas syringae* is a well‐known model bacterium for the study of plant–pathogen interactions, in part due to its remarkable broad host range (Bundalovic‐Torma et al., 2022). This hemibiotrophic bacterium can survive not only in the surface of the plant host leaves and fruits, but also in the apoplast, where it reaches through accidental wounds or natural openings, such as stomata. Within this species, there are numerous and highly diverse strains that cause a wide range of diseases in multiple economically important agronomic crops, including soybean, common bean or tomato. However, the strain‐host interaction is very specific and restricted to a very low number of plants (Mansfield et al., 2012). To overcome plant immune responses and survive within its hosts, *P. syringae* strains use different strategies, ranging from the production of compounds, such as coronatine or syringolin, to the secretion of effectors through the T3SS (Xin & He, 2013, Xin et al., 2018). This machinery along with its T3Es repertoire are absolutely required for the *P. syringae* virulence but, at the same time, they can be responsible for the infective process arresting ETI‐related responses (Cunnac et al., 2011; Mansfield et al., 2012). Thus, one of the most important means for disease management entails mobilization of plant *R* genes from non‐susceptible plants to crop commercial varieties by breeding programs (Alfano & Collmer, 2004; Boller & He, 2009).

The *P. syringae* pan‐genome, which consists of core, accessory and unique genes, encodes more than 5000 unique effector proteins grouped into 70 distinct effector families, which are certainly required for virulence and determine in most cases the host‐preference (susceptibility) for this bacterium (Dillon et al., 2019; Lindeberg et al., 2009, 2012). Recently, Laflamme et al. (2020) have constructed a *P. syringae* T3Es library (PsyTec), comprising the whole effector diversity to 494 representative alleles, to identify those T3Es able to induce ETI in the model plant *Arabidopsis thaliana*. This study found that ETI elicitation is a prominent feature of *P. syringae* effector repertoires, with nearly all analysed *P. syringae* strains carrying at least one T3E that elicit effector‐triggered responses in *A. thaliana*, with a total of 59 ETI‐eliciting alleles identified among 19 distinct T3Es families. A recent study extended this list with two additional T3Es families (Table 1) (Martel et al., 2022).

**TABLE 1:** Plants in which *Pseudomonas syringae* type III‐secreted effectors elicit effector‐triggered immunity responses. ETI: effector‐trigger immunity, HR: hypersensitive response.

Plant	ETI‐eliciting T3Es family or allele	Comment	Reference
*Arabidopsis thaliana*	AvrB AvrRpm HopA HopZ HopAR HopBJ	ETI with HR Strong ETI	Choi et al. (2021), Hockett et al. (2014), Laflamme et al. (2020), Lewis et al. (2013), Warren et al. (1998)
	AvrRpt HopO	ETI with HR Non strong ETI	Kunkel et al. (1993), Laflamme et al. (2020), Martel et al. (2022), Ruiz‐Bedoya et al. (2023), Yu et al. (1993)
	AvrE HopF HopI HopK HopX HopAA	ETI without HR Strong ETI	Gassmann et al. (1999), Laflamme et al. (2020), Ruiz‐Bedoya et al. (2023), Seto et al. (2021)
	AvrPto HopB HopD HopT HopAX HopAZ HopBA	ETI without HR Non strong ETI	Laflamme et al. (2020), Martel et al. (2022)
Bean	HopZ1a, b, Z3	ETI with HR	Rufián et al. (2018)
*Brassica napus* (canola)	HopBA1a AvrRpt2b HopI1k HopX1i HopBJ1b	Effectors producing the strongest ETI response among all tested	Breit‐McNally et al. (2022)
*Camelina sativa* (false fax)	HopA1j HopZ1a HopK1a HopBA1a HopBJ1b	Effectors producing the strongest ETI response among all tested	Breit‐McNally et al. (2022)
Tomato	AvrPto HopAB2 (N‐terminal)	Only N‐terminal domain elicits ETI for HopAB2	Ronald et al. (1992), Salmeron et al. (1994), Kim et al. (2002)
*Nicotiana benthamiana*	HopA1 HopZ1a,b, Z2, Z5 HopAU1	ETI with HR	Dahale et al. (2021), Ma et al. (2006), Zhang et al. (2022), Zhang et al. (2022)
*Nicotiana tabacum* cv *Xanthi*	HopA1	ETI with HR	Dahale et al. (2021)
*Nicotiana tabacum ´*N509´	HopAZ1	ETI with HR	Kashihara et al. (2022)
Rice	HopZ1a	ETI with HR	Ma et al. (2006)
Sesame	HopZ1a	ETI with HR	Ma et al. (2006)
Soybean	HopZ1a	ETI with HR	Ma et al. (2006)

Although most of the advances in this field have been made using *A. thaliana* as a host plant model, the obtained results could be extrapolated to other plants of agricultural interest, since the mechanisms involved in ETI are, to some extent, very well conserved among plants (Sun et al., 2020). For example, the HopZ1a effector elicits a strong ETI in *A. thaliana*, but it is also able to activate effector‐related responses in many other plants, such as *N. benthamiana*, rice, soybean, sesame, bean, and *Camelina sativa* (Breit‐McNally et al., 2022; Bundalovic‐Torma et al., 2022; Ma et al., 2006; Rufián et al., 2018). In summary, the adequate selection of these immunogenic and host‐determinant molecules is crucial for a successful strategy of a hypothetical “*à la carte*” plant vaccination.

## MEMBRANE VESICLES‐BASED RELEASE OF IMMUNOGENIC MOLECULES

Cell surface‐detached membrane vesicles (MVs) are lumen‐containing spheres of lipidic nature released to the extracellular environment by the three domains of life. Although generically termed MVs, their designation differs depending upon the taxonomic group they are produced by: MVs in Archaea and Mycobacteria; outer membrane vesicles or outer‐inner membrane vesicles in Gram‐negative bacteria; cytoplasmic membrane vesicles in Gram‐positive bacteria; exosomes, microvesicles or apoptotic bodies in Eukarya (Akers et al., 2013; Toyofuku et al., 2019; Velimirov & Ranftler, 2018). For the sake of language economy, we will adhere to a generic nomenclature and term them henceforth extracellular membrane vesicles: MVs. Such nanometre‐sized particles of single or double lipid bilayer composition range a diameter from 20 to 400 nm and participate in a manifold of biological processes, such as DNA transfer, decoy for phages and antibiotics, disposal of waste material and surface remodelling, nutrient scavenging, bacterial killing, delivery of bioactive compounds, and host immunomodulation (Flemming et al., 2023; Salvachúa et al., 2020; Toyofuku et al., 2023). To date, several routes that lead to MV formation have been elucidated including blebbing and explosive cell lysis in Gram‐negative bacteria and blebbing and bubbling cell death in Gram‐positive (Flemming et al., 2023). The stimuli known to prompt the activation of each route are diverse. Membrane blebbing results from cell envelope disturbances such imbalanced peptidoglycan biosynthesis, the accumulation of denatured proteins, antibiotic treatment, or the intercalation of hydrophobic molecules. Molecules exerting any type of stress, such as prophage‐derived holin‐endolysins, antibiotics, genotoxic agents or peptidoglycan‐degrading enzymes lead to explosive cell lysis in Gram‐negative and bubbling cell death in Gram‐positive (Furuyama & Sircili, 2021; Kulp & Kuehn, 2010; Liu et al., 2022; Toyofuku et al., 2015). Although the mechanisms underlying differential cargo packaging still remain elusive, it is well‐known that the composition of MVs is different from their parental bacterial cells, reason why these lipid‐based vectors could suppose an ancestral secretion pathway that depends on the specific packaging mechanism, the so‐called Type 0 Secretion System (T0SS) (Guerrero‐Mandujano et al., 2017).

Despite most of the research has been focused on the pathogen–mammal host models, in recent years, these membranous nanostructures are also gaining considerable attention in the field of (sustainable) agriculture given that more evidence is being gathered on their involvement in shaping plant–microbe symbiotic and pathogenic relationships in all parts of the holobiont–rhizosphere (Borrero de Acuña & Bernal, 2021). In mammal, infectious bacteria (*Escherichia coli*, *Helicobacter pylori*, *Pseudomonas aeruginosa*, or *Legionella pneumophila* to name some) MVs are overloaded with cell envelope‐associated virulence factors that manipulate the physiology of the host cell for mediating adhesion, invasion, cytotoxicity, immunomodulation, immune system elusion, actin depolymerization or formation of pores (Jan, 2017; Villageliu & Samuelson, 2022). For this reason, these extracellular nanocompartments have been considered ‘long distance weapons’ that allow the delivery of sufficient number of virulence factors to ensure its bioactivity into host tissues, a phenomenon referred to as quantal secretion (Bomberger et al., 2009; Macion et al., 2021; Rueter & Bielaszewska, 2020; Toyofuku et al., 2023). However, at the same time, these nanoparticles encompass a dual effect on target organisms, since a plethora of immunomodulatory molecules are known to be part of the MV reservoir in diverse mammal and plant pathogenic microorganisms (Ellis & Kuehn, 2010; Katsir & Bahar, 2017; Orench‐Rivera & Kuehn, 2021; Toyofuku et al., 2023; Zipfel et al., 2006).

Focusing our attention on plant pathogenic bacteria, the MVs stemmed from *Pseudomonas syringae* pv. *tomato* DC3000 and *Xanthomonas campestris* pv. *vesicatoria* contain potent immunogenic elicitors, such as the LPS‐constituent O‐antigen that acts as a decoy for the plant defensive responses while the pathogen transmits virulence factors (xylanases, proteases, and lipases) into the host cell, which have a direct impact on the degradation of the plant cell wall (Chowdhury & Jagannadham, 2013; Solé et al., 2015). In the MVs from *X. campestris* pv. *campestris* 33,913 and *X. oryzae* pv. *oryzae* PXO99, the encapsulated immunogenic EF‐Tu is perceived by its cognate immune coreceptors that bring about a drastic induction of the *Arabidopsis thaliana* defence responses (Katsir & Bahar, 2017). In another study, it was shown that MV cargo of *X. campestris* pv. *campestris* elicit broad transcriptional shifts in *A. thaliana* even at larger scale than the purified elicitors, profoundly activating the plant immune system by upregulating a manifold of immune receptors and, in consequence, the related pathways (Chalupowicz et al., 2023). In the case of *Xylella fastidiosa*, a bacterial pathogen that colonizes the xylem of important crop plants, during the infective (exploratory) lifestyle MVs act as anti‐adherence agents to prevent cellular attachment to diverse surfaces, including the walls of xylem vessels in host plants, which allows colonization of plants. Interestingly, MV secretion by *X. fastidiosa* is disrupted by the diffusible signal factor‐dependent quorum‐sensing system, which regulates the transition between this exploratory stage to the sessile lifestyle responsible for biofilm formation, xylem occlusion, and disease occurrence (Ionescu et al., 2014; Purcell & Hopkins, 1996). However, the MVs of *X. fastidiosa* encase relevant virulence factors responsible for the development of the disease which, at the same time, can also induce a hypersensitive immune response in different plants. Among which, the Type II secreted lipase/esterase LesA along with adhesins, such as hemagglutinin‐like proteins and the autoaggregation XatA autotransporter (Matsumoto et al., 2012; Nascimento et al., 2016) (Table 1).

In short, despite MVs from phytopathogenic bacteria are enriched with virulence factors, required for the full disease progression in host plants, simultaneously they can be responsible to some extent for infection blocking, since these lipidic vehicles are overloaded with immunogenic molecules able to trigger MTI‐related responses. Emerging from these findings arise important biotechnological questions, could plants be exposed to MVs prior phytopathogenic infection to generate a sort of plant immunity? Might these MV‐mediated defence responses be improved by molecular engineering?

## FUTURE PERSPECTIVES AND CONCLUDING REMARKS: ENGINEERING MEMBRANE VESICLES WITH TYPE 3 EFFECTORS FOR AN IMPROVED PLANT VACCINATION

**FIGURE 3:**
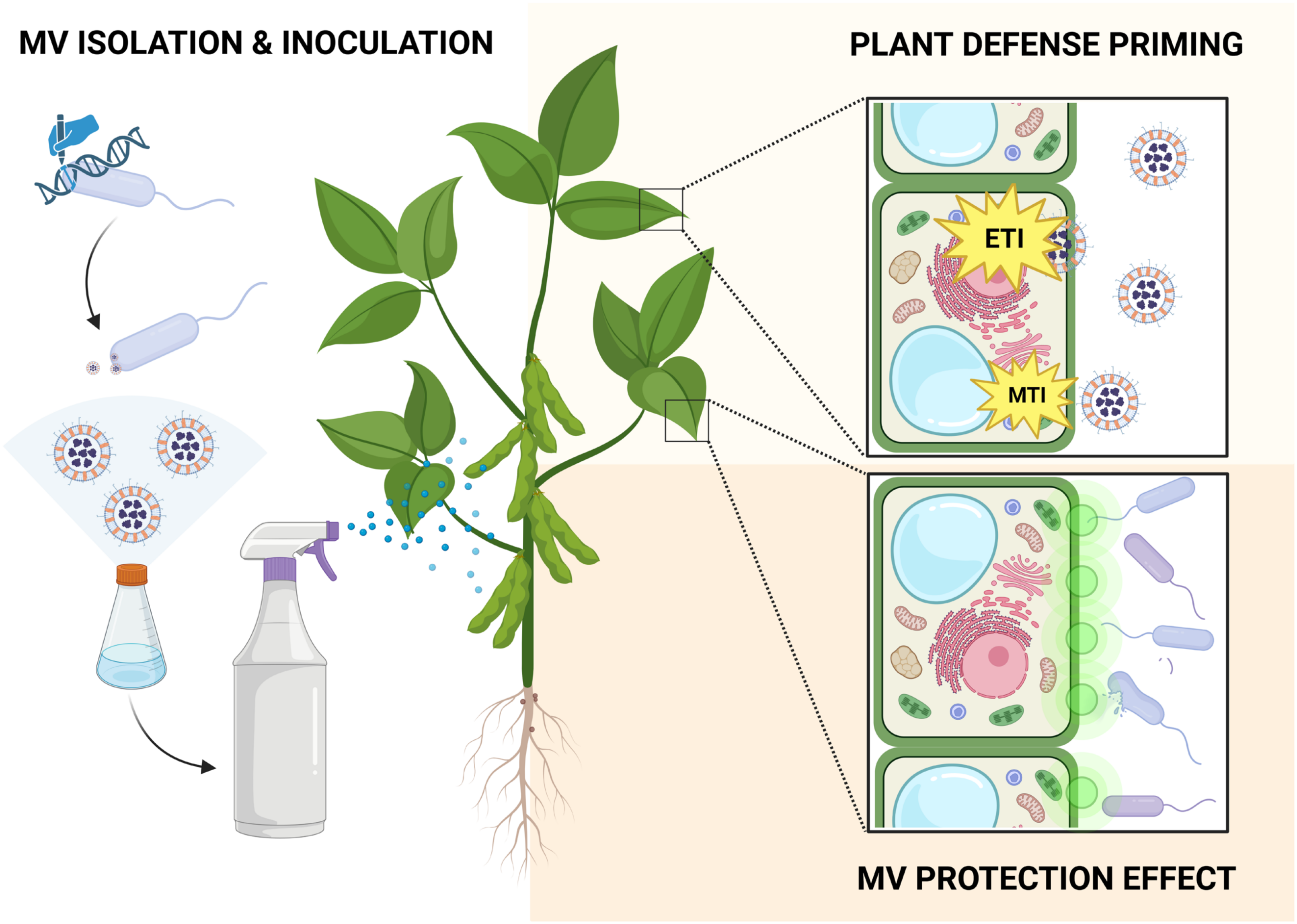
Generic strategy envisioned to apply “*à la carte*” engineered membrane vesicles (MV) encasing appropriate type III effector (T3E) to prime plants for subsequent crop infestation. Isolation and application of T3E‐enriched MVs is presumed to boost host immunity, rendering the plant vaccinated against upcoming infections, in a sort of organism‐free plant vaccination. Created with BioRender.com.

MV isolation and quantification technologies and procedures have been optimized at an unprecedented pace in the recent years. Nowadays, cutting‐edge procedures encompass the use of a series of purification steps involving ultrafiltration, ultracentrifugation, and fraction separation by density gradients coupled with quantification and visualization techniques, such as electron microscopy, flow cytometry, fluorescence‐ (lipid‐dye), and scattering light‐reliant (Nanosight) detection of MVs (Baeza et al., 2021). These technologies have eased considerably the isolation of pure MV fractions from all sorts of bacteria, archaea, and eukaryotes (Toyofuku et al., 2023). Thus, one could benefit from these technologies to purify (i) naturally produced immunogenic molecules‐loaded MVs by *P. syringae* and akin plant pathogens and (ii) “*à la carte*” engineered MVs endowed with host‐determinant T3Es of this bacterium. In this context, we propose to employ engineered MVs as a platform for the generation of organism‐free plant vaccines. Thus, upon the application of engineered MVs onto the plant leaves, the selected T3Es will be efficiently delivered into the lumen of the vegetal cells, triggering strong ETI responses on selected plants as shown elsewhere (Cai et al., 2023; McMillan et al., 2021; Wang et al., 2023). In this manner, boosting the host MAMP‐ and effector‐triggered immunities will render the plant vaccinated against upcoming infections by a plethora of phytopathogens, since both defence responses can confer long‐lasting resistance against different bacteria (Cai et al., 2019; Durrant & Dong, 2004) (Figure 3). Additionally, since the MVs are quantal delivery systems which transport individual T3Es through large distances (Toyofuku et al., 2023), these MV‐based approach could be considered specially efficient for plant vaccination because (i) the concentration of bioactive molecules is elevated within the MVs thereby fostering their incorporation into the target cell to a larger scale than by free diffusion and ensuring their biological activity, and (ii) the absence of the remaining T3E arsenal avoid the potential subversion of the plant immune responses that are often collectively mediated by these effectors (Toyofuku et al., 2023).

Furthermore, specific immunogenic T3E domains could be selected instead of using the entire effector protein for MV engineering, thereby increasing the eliciting T3E ability and reducing various biotechnological risks, such as the difficulties of translocating the full‐length T3E protein into the MV, or even avoiding the presence of other T3E domains that might be involved in the suppression of ETI responses. For instance, the N‐terminal domain of the HopAB2 effector elicits ETI in resistance tomato cultivars, whereas the C‐terminal domain is an E3 ubiquitin ligase that suppresses this response by targeting the tomato kinase Fen for its degradation (Janjusevic et al., 2006; Lo et al., 2017; Rosebrock et al., 2007). Obviously, selection of the appropriate T3E candidate for a given plant is critical to elicit a strong and long‐lasting defence priming. In this regard, diverse characteristics, such as the T3E size, the plant response degree elicited, and the range of plants in which the T3E activates an ETI, must be taking into consideration. As an example, HopZ1a could be a suitable T3E, since it is not a large T3E and activates a strong HR in a wide range of plants.

**FIGURE 4:**
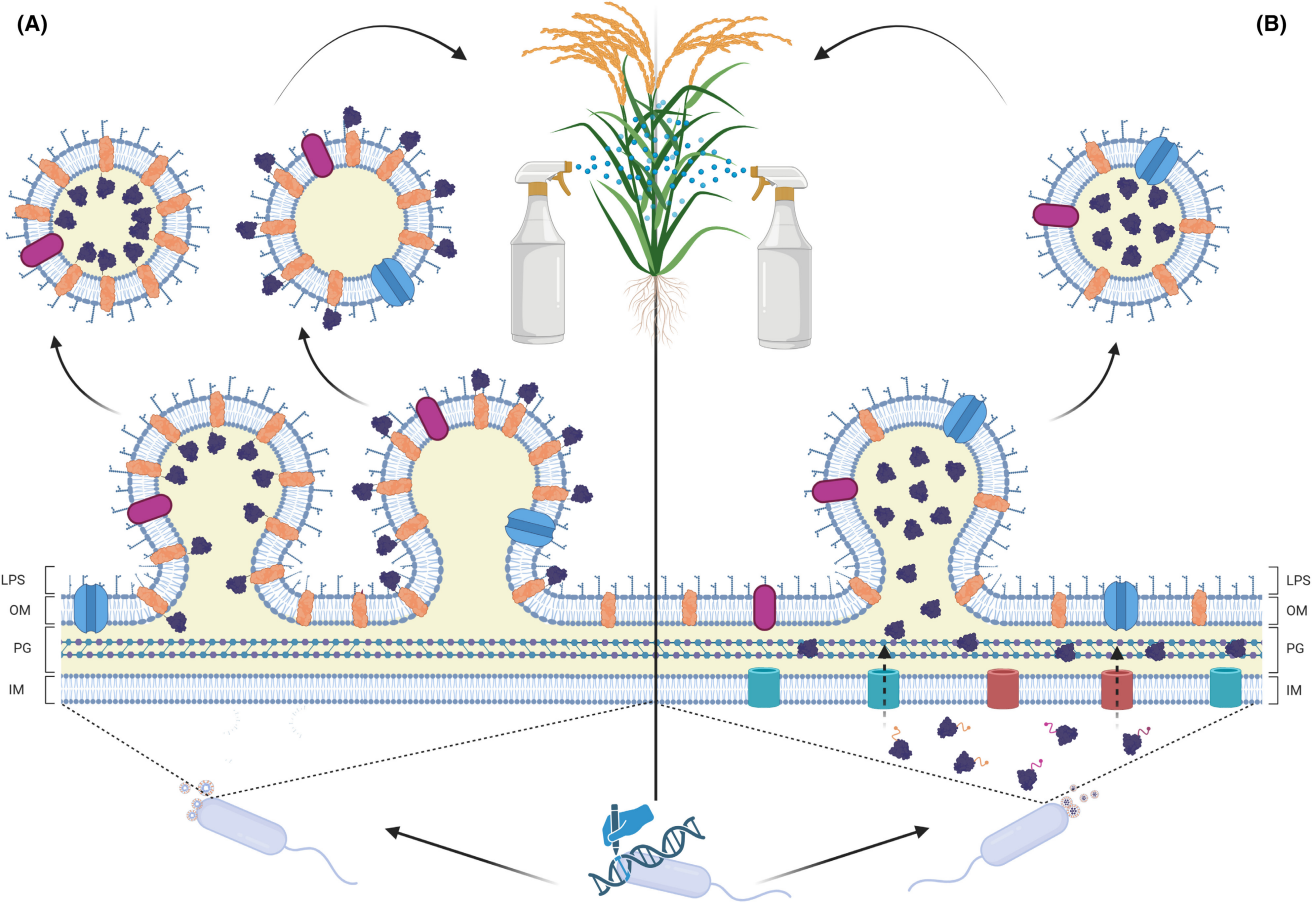
Strategies for Gram‐negative bacteria membrane vesicle (MV) engineering. (A) Generation of chimeric proteins comprising constituents of the MV scaffold protein fused to the type III effector (T3E) to ensure co‐transport into the MVs. (B) Production of recombinant proteins fused to a signal peptide recognizable by membrane translocases to compel its transport into the periplasmic space and its packaging into the MVs. Created with BioRender.com.

Purification of naturally occurring MVs from plant pathogens is a straightforward process but engineering the MV cargo to introduce T3Es as constituents of its proteinic architecture might be more challenging. To the end of modifying MV cargo, we envision a protein engineering strategy. On the one hand, the proteinic scaffold components of the MVs, as well as proteins promoting MV biogenesis should be identified by appropriate proteomic analyses. It is well known that in most Gram‐negative bacteria proteins anchoring the peptidoglycan to the outer membrane or cytoplasmic proteins – unless the MV stems from cell lysis are poorly represented in the MV proteome (Lappann et al., 2013; Toyofuku et al., 2023). Conversely, periplasmic proteins, such as beta‐lactamases, and outer membrane porins (OprF, OmpT, OmpU) are significantly abundant in OMVs of Gram‐negative bacteria (Cassin & Tseng, 2019; Ciofu et al., 2000; Schaar et al., 2011; Tiku et al., 2021; Wessel et al., 2013; Zingl et al., 2021). In fact, the inter‐species conserved OprF porin seems to be overrepresented in MVs and its truncation leads to a diminishment in OMV yield (Wessel et al., 2013). We envisage two previously tested strategies for MV engineering for Gram‐negative bacteria: (i) the generation of chimeric proteins consisting of the MV scaffold protein fused to the protein of interest to ensure co‐transport into the MVs, and (ii) the generation of recombinant proteins fused to a signal peptide to be translocated into the periplasmic space and packaged into the MVs (Figure 4) (Dammeyer et al., 2011; Shi et al., 2021). For the first strategy, chimeric proteins consisting of the MV‐scaffolding proteins fused to the selected T3E can be generated. We suggest the use of abundant membrane‐anchored proteins, such as the above‐mentioned porins, to ensure directed protein co‐transport into MVs. T3SS effectors can be C‐ or N‐terminally fused or inserted into permissive sites – domains whose alteration does not affect overall protein topology of the selected membrane‐residing protein to flip its orientation from the inner to the outer leaflet of the MVs, depending on the user's aim (Thanvi et al., 2023; Wong et al., 1995). The topology of each membrane‐residing protein of choice must be evaluated on case to case basis. Outer exposure of the protein might be crucial for its recognition by cognate plant receptors, but it can hinder its import into the target cell due to MV‐lipid rafts and cholesterol‐rich membrane microdomains fusion dynamics (Gurung et al., 2011; Kaparakis‐Liaskos & Ferrero, 2015; Prados‐Rosales et al., 2011; Söderblom et al., 2005; Tulkens et al., 2020). We propose the use of a flexible linker between both proteins to prevent detrimental steric effects and of a protease recognition site only cleavable by innate plant proteases placed up‐ or down‐stream the flexible linker that could aid to release the T3E from the integral membrane protein and thereby allow its freely diffusion throughout the plant cell (Asai & Shirasu, 2015; Huehls et al., 2015). In fact, certain T3E are self‐cleavaged after being secreted from bacteria and delivered into host cells (Lewis et al., 2009). This feature could provide significant advantages for the T3E release from the carrier MV and may facilitate the ETI elicitation.

The second strategy entails the overproduction of recombinant T3Es fused to a signal peptide recognizable by classical secretion systems (SecYEG and Twin arginine) to compel its transport into the periplasmic space (Freudl, 2018; Kaushik et al., 2022; Natale et al., 2008). After translocation, the signal peptide is cleaved, and the effector can be randomly packed into the MV content. The sorting mechanism for MV packaging is still unknown but it is expected that heavily boosting the abundance of a protein in the periplasmic fraction forces a partial incorporation of the overall protein content into the MV. However, the selection of signal peptides to encapsulate proteins of interest within the MVs should not only be restricted to those relying on classical translocases. For instance, novel research has proven that an alpha helix‐containing vesicle nucleating peptide (termed VNp) orchestrates MVs formation in *E. coli* without affecting its growth (Eastwood et al., 2023). Remarkably, when a protein of interest is C‐terminally spliced to VNp its previous cytoplasmic fate is altered resulting in its massive incorporation into the arising MVs. Whether this observation is applicable to other biotechnologically relevant strains remains to be determined. Clearly, further bioinformatic mining and in vivo assays are required in this field toward the discovery of novel MV‐specific targeting peptides.

In conclusion, the development of MV‐based molecular inoculants have great biotechnological implications in the agriculture field, avoiding soil and water contamination concomitant to the use of chemicals. The benefits of this organism‐free biocontrol agent/or plant vaccine go beyond the avoidance of chemical inputs since it also bypasses the problematic use in Europe of genetically modified organisms. This unrestrained biotechnology alternative offers a green option for the must‐needed sustainable agriculture, preventing severe crop‐associated diseases that burden economy and solving to some extent the problem of hunger. Beyond that the impact of MV‐engineering transcends the agricultural research being of grand interest in other fields including bioremediation and biomedicine with applications for vaccine production, drug delivery, and the production of valuable chemical products (Baker et al., 2014; Kim et al., 2015; Su et al., 2017).

## AUTHOR CONTRIBUTIONS

**Irene Jiménez‐Guerrero:** Conceptualization (supporting); visualization (lead); writing – original draft (equal); writing – review and editing (supporting). **Francisco Javier López‐Baena:** Funding acquisition (equal); writing – original draft (supporting); writing – review and editing (supporting). **José Manuel Borrero‐de Acuña:** Conceptualization (equal); funding acquisition (equal); writing – original draft (equal); writing – review and editing (lead). **Francisco Pérez‐Montaño:** Conceptualization (equal); funding acquisition (equal); writing – original draft (equal); writing – review and editing (lead).

## FUNDING INFORMATION

This work was funded with grants PID2019‐107634RB‐I00 funded by MCIN, PID2020‐118279R funded by MCIN/AEI/10.13039/501100011033 and by ‘ERDF A way of making Europe’; PID2021‐122395OA‐I00 funded by MCIN/AEI/10.13039/501100011033; TED2021‐130357B‐I00, funded by MCIN/AEI/10.13039/501100011033 and by ‘European Union NextGenerationEU/PRTR’; ProyExcel_00450 and EMERGIA20_00048, funded by Junta de Andalucía (Spain).

## CONFLICT OF INTEREST STATEMENT

None declared.
